# *Ficuserecta* Thunb Leaves Alleviate Memory Loss Induced by Scopolamine in Mice via Regulation of Oxidative Stress and Cholinergic System

**DOI:** 10.1007/s12035-021-02358-1

**Published:** 2021-04-01

**Authors:** Eunjin Sohn, Yu Jin Kim, Joo-Hwan Kim, Soo-Jin Jeong

**Affiliations:** 1 Clinical Medicine Division, Korea Institute of Oriental Medicine, Daejeon, 34054 South Korea; 2 Department of Life Science, Gachon University, Seongnam, 21936 South Korea

**Keywords:** Acetylcholinesterase, *Ficus erecta* Thunb leaf, Memory impairment, Neurodegenerative diseases, Nrf2/HO-1, Oxidative stress, Scopolamine

## Abstract

We examined the neuropharmacological effects of ethanol extract of *Ficus erecta* Thunb leaves (EEFE) on cognitive dysfunction in a scopolamine (SCO)-induced memory impairment animal model. Memory impairment was measured using the Y-maze test and passive avoidance task (PAT). For 19 days, EEFE (100 or 200 mg/kg) was treated through oral administration. Treatment with EEFE ameliorated memory impairment in behavioral tests, along with significant protection from neuronal oxidative stress and neuronal cell loss in the brain tissues of SCO-injected mice. Antioxidant and neuroprotective effects of EEFE were further confirmed using in vitro assays. Our findings indicate that the mechanisms of neuroprotection and antioxidation of EEFE are regulated by the cholinergic system, promotion of cAMP response element-binding protein (CREB) phosphorylation, and the nuclear factor erythroid-2-related factor 2 (Nrf2)/heme oxygenase (HO)-1 signaling activation. The current study proposes that EEFE could be an encouraging plant resource and serve as a potent neuropharmacological drug candidate against neurodegenerative diseases.

## Introduction

Alzheimer’s disease (AD) is a devastating neurodegenerative disease with aging-related cognitive and memory dysfunction. Neurodegeneration in AD is characterized by synaptic injury, followed by neuronal death that causes cognitive decline and memory loss [1, 2]. Oxidative damage plays a crucial part in the pathogenesis of AD, and neuronal degeneration is engaged in oxidative damage to all bio-macromolecule types in AD patients [3, 4]. Reactive oxygen species (ROS) are likely to intensify the disease progression by oxidative stress and are the hallmarks of AD even in the early phases [5].

Cholinergic system dysfunction is the most critical neurotransmitter system related to memory and learning, which degenerates first in the early stages of AD [6]. Increasing reports indicate that neurodegenerative diseases such as AD are involved in enhanced oxidative stress and disorders of the cholinergic neurons. Although cholinergic agonists, such as tacrine (TAC) [7, 8] and donepezil [9], are the approved pharmacological treatment for AD, they have restrictions in the form of severe toxic side effects and shorter half-lives. Therefore, attention has been focused on probing for phyto-antioxidants for the treatment or prevention of AD through their ability to defend antioxidant capacity and strengthen cholinergic function [10]. Many studies showed that the intake of natural extracts from plants has neuroprotective abilities such as memory enhancement, learning, and cognitive functions due to the capacity to protect neuronal cells from injury induced by neurotoxins and oxidative stress [11, 12]. Therefore, complementary and alternative medicines for treatment of AD are highly needed to be synthesized utilizing natural plant sources.

*Ficus erecta* Thunb (*F. erecta*) is a tree or deciduous or semideciduous shrub that is harvested from the wild for local uses as foods, sources of fiber, or as ornaments due to the resistant properties towards the fungus [13, 14]. Especially, the wild fig species *F. erecta* is distributed across the southern seashore areas of Korea and East Asia, including Japan and Vietnam [13, 15]. In addition, *F. erecta* has active medicinal properties and is used for the remedy of inflammatory diseases. Yoon et al. suggested that *F. erecta* leaves have anti-osteoporotic activity through reduced differentiation into osteoclasts and inhibiting induction of osteoporotic inflammatory factors *in vitro* [16]. Recently, another report suggested that extracts from the fruits of *F. erecta* exert antioxidant and antithrombotic activities [17]. In spite of phytotherapy and pharmacological activities of *F. erecta*, its beneficial effects in a neuronal context have not been fully investigated.

Therefore, the current study aimed to assess neuroprotective effects of the ethanol extract of *F. erecta* leaves (EEFE) in a scopolamine (SCO)-induced memory impairment animal model. SCO, muscarinic acetylcholine receptor antagonist, interferes with the cholinergic system in the brain, leading to memory impairments in humans and rodents, and is an useful experimental model to evaluate the neuroprotective effects [8, 10, 18]. As mentioned above, the FDA-approved medications for AD mainly target acetylcholinesterase (AChE) activation or *N*-methyl-d-aspartate (NMDA) receptor. Many recent types of research have tried to develop novel anti-AD drugs to target amyloid-ß (Aß) [19–21] according to the Aß hypothesis [22], but their approaches unfortunately have failed in clinical trials. What is the limitation or problem of the drugs to treat AD? Based on all of the above, single-molecular targeting may be unsuitable for developing AD treatment because of the complexity of the AD pathogenesis compared to other diseases [23, 24]. If so, what is the best scientific approach to understanding the complexity of AD and developing suitable medications? We may need to consider the efficacy of targeting the major biomolecule as well as controlling the overall phenomenon of AD such as cognitive impairment, neuronal death, and neuroinflammation. Actually, recent trends in new drug development have shifted to multiple targeting rather than single-molecular targeting [25, 26]. Cocktail therapy mixing various active compounds is thought to be one of proper manner [27]. Additionally, natural plant is a mixture of various compounds and an important material in developing multiple targeted therapies. In this regard, we have utilized a medicinal plant *F. erecta* in the AD drug research. This study highlights the role of efficacy of natural product *F. erecta*, as a new effective candidate in the treatment of memory impairment, by using behavioral test [28] and we performed simultaneous determination of *F. erecta* to determine the standard compounds. Moreover, we investigated possible mechanisms of EEFE to regulate neuronal dysfunction and damage such as changes in the cholinergic system, oxidative stress levels, and the memory-related proteins in brain tissues.

## Methods and Materials

### Plant Material

The dried leaves of *F. erecta* were collected in Gasiri, Pyoseon, Seogwipo, Jeju, Korea in September 2017 and identified by Professor Joo-Hwan Kim (Gachon University, Seongnam, Korea). A voucher specimen (SCD-A-114) was deposited at the Clinical Medicine Division, Korea Institute of Oriental Medicine (Daejeon, Korea).

### Preparation of EEFE

The dried leaves of *F. erecta* (3.4 kg) were extracted twice with 60 L aqueous ethanol using an electric extractor (COSMOS-660, Kyungseo Machine Co., Incheon, Korea) for 3 h at 80±2 °C. The filtered extract solution was concentrated in a 20 L round-bottle flask using a rotary vacuum evaporator (EV-1020, Daihan Scientific Co., Wonju, Korea), and then freeze-dried to generate powdered extracts (636.13 g). The yield of EEFE was 18.71%.

### Animal Grouping and EEFE Administration

Seven-weeks-aged male ICR mice were obtained from the Daehan Biolink (Cheongju, Korea) and acclimated for 1 week prior to the study with standard foods and water supplied ad libitum in individual acryl cages. All mice were kept in under 12-h light/dark cycle at room temperature 22±2 °C, and 55% humidity-controlled conditions. The animal study began to 8-weeks-aged (weight, ~30 g) and animals were monitored for 19 days. For the grouping of animals, ICR mice were assorted into five groups (*n*=8/group). The normal (NOR) group received an equal volume of vehicle (distilled water, DW). The SCO group was induced by a single injection of SCO (1 mg/kg, i.p.) for memory deficit. The EEFE group was treated EEFE to concentrations of 100 mg/kg (EEFE-100) and 200 mg/kg (EEFE-200) by oral administration. For the positive control, the TAC group was administered TAC dissolved in DW (USP, Rockville, MD, USA) (10 mg/kg). All mice underwent behavioral tests from the 12th to the 15th day. After oral administration of EEFE or TAC solution, mice were injected with SCO (i.p.) within 60 min. The NOR group received saline 30 min after treatment with DW. The experimental protocol was modified based on the previous studies [28, 29]. To determine the dosage of EEFE in mice, we considered that the usual dosage of natural plant is approximately 5–30 g/human adult/day of the raw natural plant in clinical application [30] and calculated animal dose range from 80 to 500 mg/kg in mice using the animal equivalent dose by human equivalent dose (HED). The EEFE dosage was applied 100 or 200 mg/kg/day. No treatment-related clinical signs, body weight loss, and animal death were observed during the experimental period in all animals. Animal experiments were performed under a non-toxic concentration of EEFE. The experiments were performed according to the National Institutes of Health (NIH) guide for the care and use of laboratory animals and approved by the Korea Institute of Oriental Medicine Institutional Animal Care and Use Committee (IACUC Approval No.18-002, 9 Feb. 2018). These animal study procedures were performed according to Animal Research: Reporting of In Vivo Experiments (ARRIVE) guidelines.

### Morphologic Analysis

At the end of the experimental period, mice from each group were sacrificed under deep anesthesia. Three mouse brains from each group were perfused transcardially with saline and then fixed in 4% paraformaldehyde. Each of the hippocampal and cortical tissues isolated from five mice of each group was immediately stored at −80 °C until further analysis. Brain paraffin blocks were sliced into 4-μm-thick sections. The slides were deparaffinized and hydrated with xylene and sequentially of ethanol solutions, respectively. Slides were immersed in 1% cresyl violet acetate solution for Nissl staining, washed with water, and dehydrated with 90% and 100% ethanol for 5 min before mounting in xylene. Mounted slides were then captured at × 400 magnification using a microscope (Olympus DP71, Tokyo, Japan). Image analysis was performed by blinded investigators using the Image J software program (Java-based image processing program, NIH, Bethesda, MD, USA).

### Immunoblotting Analysis

Isolation of nuclear and cytosolic proteins was performed with a Nuclear Extraction Kit (Cat. 78833, Thermo Fisher Scientific, Waltham, MA, USA). Nuclear and cytosolic fractions were obtained according to the manufacturer’s protocol. Briefly, homogenized tissues were centrifuged at 300×*g* for 5 min at 4 °C. The pellets were mixed with hypotonic buffer; 10% Nonidet P-40 assay reagent was added to the pellet. Nuclear and cytoplasmic extracts were obtained by centrifugation at 14,000×*g* for 30 s natant and stored at −80 °C until use. Whole-brain lysed tissue with protein lysis buffer (pH 7.5, RIPA, Cat. 89900, Thermo Fisher Scientific) were resolved on polyacrylamide gels and then transferred on 0.2-μm polyvinylidene fluoride (PVDF) membranes using the Trans-Blot transfer system (Bio-Rad, Hercules, CA, USA). The blocked membranes were probed with primary rabbit anti-total cAMP response element-binding protein (CREB) (Cat. ab178322, AB_2827810, Abcam, Cambridge, UK, phospho-CREB (pCREB) (Cat. ab32096, AB_731734, Abcam), choline acetyltransferase (ChAT) (Cat. SC-55557, AB_2291743, Santa Cruz Biotechnology Inc., Dallas, TX, USA), nuclear factor erythroid-2-related factor 2 (Nrf2) (Cat. PA5-27882, AB_2545358, Thermo Fisher), heme oxygenase 1 (HO-1) (Cat. ab85309, AB_2118656, Abcam), mouse anti-ß-actin (Cat. A5316, AB_476743, Sigma-Aldrich, Saint Louis, MO, USA), tubulin (Cat. ab6160, AB_305328, Abcam), or nucleolin (Cat. ab22758, AB_776878, Abcam. Washed membranes with tris-buffered saline with 0.1% tween 20 (TBST) were incubated with secondary antibodies anti-rabbit- horseradish peroxidase (HRP). Immuno-reactive membrane was developed using the chemiluminescent substrate reagents (Cat. 34577, Amersham Bioscience, Piscataway, NJ, USA). Protein bands were obtained by analyzing the captured signals using an imaging analyzer (ChemiDox, Las-4000 MINI, Fuji photo, Tokyo, Japan).

### Acetylcholine (Ach) Level and AChE Activity Measurement

ACh levels (Cat. E4453, Biovison, Milpitas, CA, USA) and AChE (Cat. MBS2019857, MyBioSource, San Diego, SC, USA) activity in brain lysates were measured according to commercial manufacture’s protocols. The brain homogenates were applied onto the incubated 96-well plates and measured for the absorbance of the mixture using a microplate reader (Benchmark Plus, Bio-Rad) at 450 nm.

### Measurement Superoxide Dismutase (SOD) Activity, and Glutathione (GSH) and Malondialdehyde (MDA) Levels in Brain Tissue

Intracellular SOD activity (Cat. MBS034842, MyBioSource), GSH (Cat. KA0797, Novos Biologicals, Centennial, CO, USA) level and MDA contents (Cat.10009055, Cayman, Ann Arbor, MI, USA) in brain tissues were measured according to the commercial manufacturer’s protocols. The enzyme activity and levels in supernatants separated from brain lysates were analyzed. Absorbance of reaction mixture in 96-well plate was measured using a microplate reader (Benchmark Plus, Bio-Rad).

### Animal Behavioral Tests

As we have described previously [28], the memory and learning function of experimental mice were assessed using Y-maze and PAT. All efforts were made to minimize animal suffering. The PAT was performed using an electronic shock generator with a lighted and darkened compartment (Jeungdo Bio & Plant Co. Ltd., Seoul, Korea). Transfer latency time was recorded which mice remain to time in the lighted compartment within 5 min. Y-maze test was measured by counting the number of spontaneous alternations using a tracking system software (EthoVision, Noldus Information Tech, Wageningen, Netherlands). The mice were initially placed in one arm and were then allowed to explore freely to enter the other arms in sequence (e.g., ABC, BCA, or CAB) for 8 min. After 30-min SCO injection, the behavioral tests were started, and mice were orally treated with EEFE prior to the SCO injection.

### Radical Scavenging Activity Assay

2,2′-Azinobis [3-ethylbenzothiazoline-6-sulfonic acid]-diammonium salt (ABTS) and 2,2-diphenyl-1-picrylhydrazyl (DPPH) radical scavenging activity were measured according to the previously described method [31]. Aliquots of EEFE solution (100 μL) at various concentrations were mixed with 100 μL ABTS^+^ solution or DPPH solution of 0.15-mM concentration in methanol, respectively. The reaction mixture was incubated for 5–30 min in the dark at room temperature. The absorbance of the resulting solution was measured at 734 or 517 nm with a microplate reader (BioTek Instruments, Winooski, VT, USA), respectively. The radical scavenging capacity of each tested sample was represented as a scavenging activity (%).

### Cell Culture and Cytotoxicity Assay

Mouse hippocampal neuronal HT22 cell line (Cat. SCC129, CVCL_0321, Sigma-Aldrich) was investigated throughout this study. HT22 cells were maintained in Dulbecco’s modified eagle’s medium (Hyclone/Thermo Fisher Scientific) with 10% fetal bovine serum (Hyclone/Thermo Fisher Scientific) and penicillin/streptomycin in a 5% CO_2_ incubator at 37°C. Cells were plated onto 96-well culture plates at a density of 5 × 10^3^/well and treated with EEFE. HT22 cells were co-treated with hydrogen peroxide (H_2_O_2_, Cat. 88579, Sigma-Aldrich) at a concentration of 250 μM and various concentrations of EEFE. For the determination of cell viability, a cell counting solution (CCK-8, Cat. CK04, Dojindo, Kumamoto, Japan) was added to each well, and then plates were incubated for 4 h in a CO_2_ incubator. The optical density was measured at 450 nm on a microplate reader (BioTek Instruments). Lactate dehydrogenase (LDH) leakage in a medium is an indicator of cytotoxicity. Therefore, LDH assay was performed using a colorimetric alternative to radioactive cytotoxicity assay kit (Cat. G1780, Promega, Madison, WI). Briefly, plated cells were lysed and prepared lysates and supernatants to induce maximal LDH release and experimental LDH release, respectively. After 30 min of incubation at room temperature in dark, absorbance was measured in a microplate reader at 490 nm (Molecular Device, Spectramax i3, San Jose, CA, USA). The same volume of blank medium was used as the background control.

### High-Performance Liquid Chromatography (HPLC) Analysis

#### Chemicals and Reagents

Rutin and chlorogenic acid were purchased from ChemFaces Biochemical (Wuhan, China), and kaempferol-3-O-rutinoside was obtained from Biopurify Phytochemicals (Chengdu, China), respectively. These compounds were analyzed for a purity of ≥98.0% by HPLC. The HPLC grade acetonitrile and water of HPLC grade (J. T. Baker Chemical, Phillipsburg, NJ, USA), and TFA reagent (Sigma-Aldrich) were purchased. 

#### Preparation of Sample and Standard Solutions

The powdered EEFE was dissolved in 80% aqueous methanol to a final concentration of 10 mg/mL and filtered through a 0.45-μm-pore-size syringe filter for HPLC analysis. The stock solutions of three standard compounds were melted with methanol to a final concentration of 1.0 mg/mL. Each stock solution was diluted with methanol to a final concentration of 0.1 mg/mL before HPLC analysis.

#### Chromatographic Conditions

 Waters Alliance e2695 HPLC system (Waters Corp., Milford, MA, USA) equipped with a photodiode array (PDA) detector (#2998; Waters Corp) was used for HPLC analysis. The data were acquired and processed using Empower software (version 3; Waters Corp). The three standard compounds were separated on Sunfire C18 analytical column (250×4.6 mm, 5 μm) (Waters Corp) maintained at 35 °C. The mobile phases consisted of two solvents 0.1% (v/v) aqueous TFA (A) and acetonitrile (B). The gradient conditions were 10–23% B for 0–30 min, 23–100% B for 30–40 min, and 100% B for 41–50 min. The flow rate and injection volume were 1.0 mL/min and 10 μL, respectively. The ultraviolet (UV) wavelength range of the PDA detector was 190 to 400 nm.

### Statistical Analysis

All data were expressed as the mean ± SEM. A value of *p* < 0.05 was considered to indicate statistical significance. Prism software (Graph Pad, version 8.4.1, San Diego, CA, USA) was used for all analyses. Statistically significant differences were evaluated with one-way analysis of variance (ANOVA) for comparison three or more groups, and with an unpaired or paired Student’s *t-*test for comparison between two groups. All experiments were performed individually at least three times.

## Results

### Ameliorating Effects of EEFE on Memory Impairment in SCO-Treated Mice

**Fig. 1:**
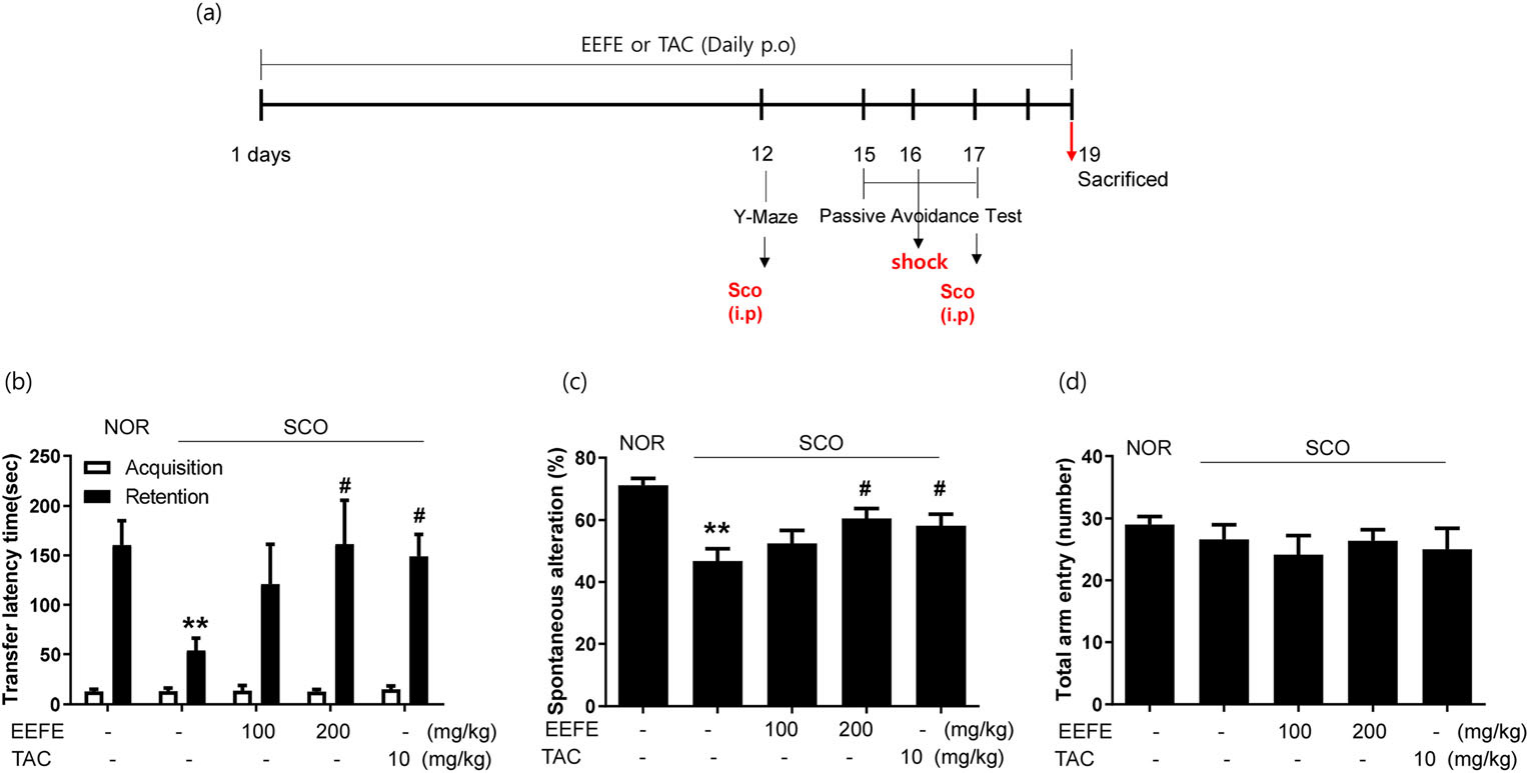
Effects of EEFE on memory impairments in SCO-injected mice. **a** Schematic description of the experimental timeline. **b** For the passive avoidance test (PAT), an acquisition trial was first performed, and a retention trial was conducted for 300 s at 24 h after the acquisition trial. **c**, **d** For the Y-maze test, the spontaneous alternation of behavior (**c**) and the number of total arm entries were monitored during an 8-min session (**d**). TAC was used as a positive control. Data are presented as the mean ± SEM (*n*=8). ^**^p<0.01 vs NOR group, ^#^*p*<0.05 vs SCO group. EEFE, ethanol extracts of *Ficus erecta*; NOR, normal; SCO, scopolamine; TAC, tacrine

Figure 1a presents a time plan for the animal experiments. For the induction of memory impairment, SCO was administered to the mice [29]. To investigate whether the effect of EEFE enhances the restoration from memory impairments, we performed PAT and Y-maze tests in the SCO-induced cognitive deficit mice. In PAT, the SCO group exhibited a marked reduction in transfer latency time compared with the NOR group (Fig. 1b, *p*<0.01). However, EEFE treatment improved the latency time reduced by SCO injection compared to the EEFE at 200 mg/kg administration (Fig. 1b). The deficit by SCO injection in spontaneous alternations of the Y-maze test was also significantly reversed in the EEFE-200 group compared with the SCO group (Fig. 1c). The number of arm entries among the experimental groups was not observed a significant difference (Fig. 1d). The TAC group, a positive control [8], also exhibited marked attenuation of SCO-induced memory impairment in both PAT and Y-maze tests.

### Neuroprotective Effects of EEFE in SCO-Induced Memory Impairment Mice and H_2_O_2_-Stimulated HT22 Hippocampal Cells 

**Fig. 2:**
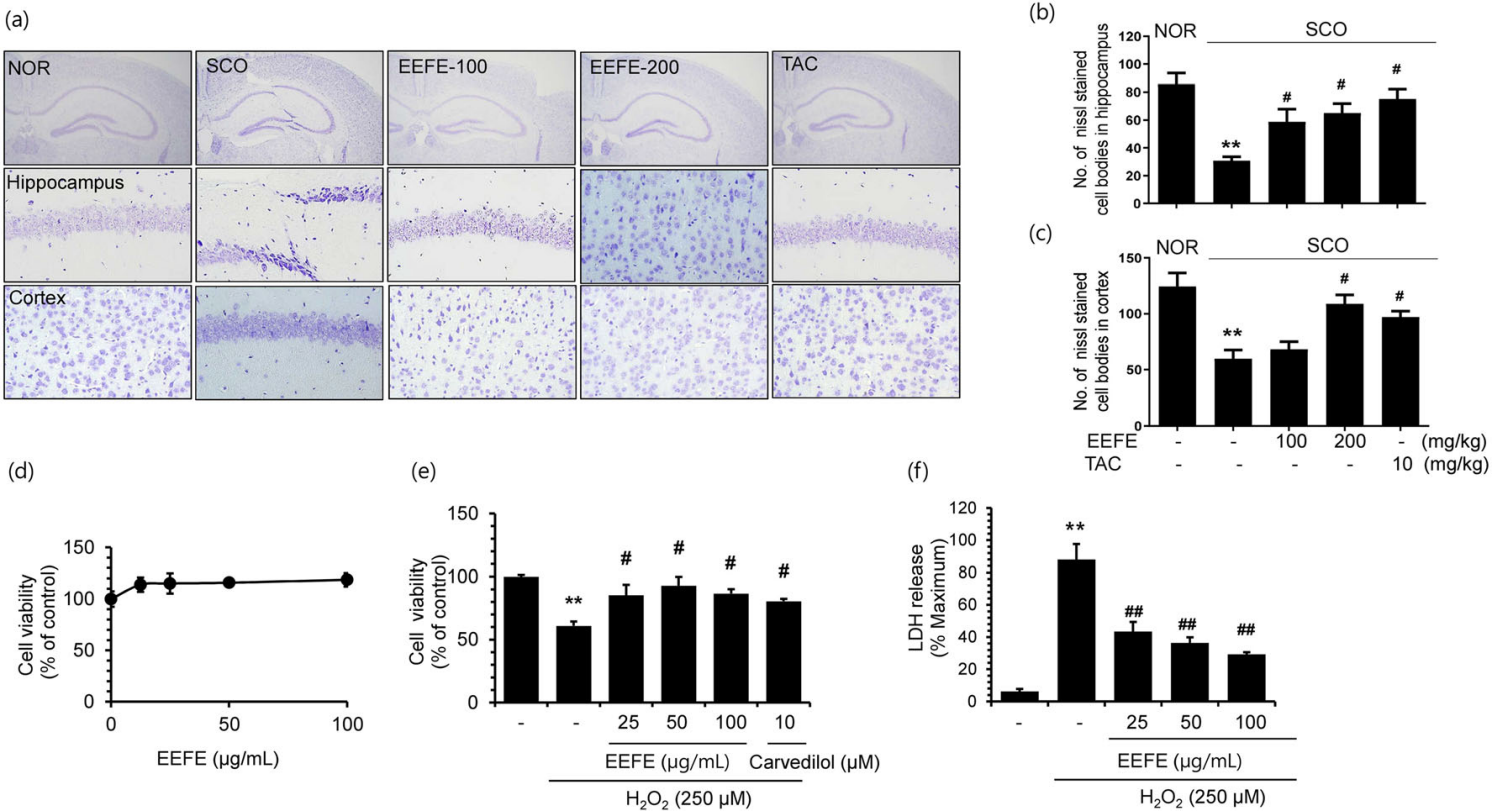
Effects of EEFE on neuronal damage in SCO-induced memory impairment mouse brains and H_2_O_2_-treated neuronal cells. **a** Sections of the hippocampus and cortex were prepared for Nissl staining using cresyl violet solution. Representative photomicrographs were captured at magnifications of ×400. **b**, **c** The graphs show the number (No.) of Nissl stained cells in the CA1 of the hippocampus (**b**) and cortex (**c**). TAC was used as a positive control. Data are presented as the mean ± SEM (*n*=3). ^**^p<0.01 vs NOR group and ^#^*p*<0.05 vs SCO group. **d** HT22 hippocampal cells were treated with EEFE for 24 h to examine the cytotoxicity using CCK assay. **e**, **f** HT22 hippocampal cells were exposed to H_2_O_2_ in the absence or presence of EEFE for 6 h. Cell viability and toxicity were determined using the CCK (**e**) and LDH release (**f**) assays. Carvedilol was used as a positive control. The results of three independent experiments are expressed as mean ±SEM (*n*=3). ^**^p<0.01 vs untreated control, and ^#^*p*<0.05 or ^##^*p*<0.01 vs H_2_O_2_-treated cells. EEFE, ethanol extracts of *Ficus erecta*; NOR, normal; SCO, scopolamine; TAC, tacrine; CA1: Cornu Ammonis 1; H_2_O_2_: hydrogen peroxide

To investigate whether the EEFE has protective effects against neuronal injury, we examined neuronal cell morphology using Nissl staining to image both the hippocampal and cortex areas of the mouse brain. In the SCO group, neuronal cell changes, such as neuronal cell loss, damage, and shrinkage, were markedly showed in the hippocampal and cortex region in comparison to the NOR group. In contrast, EEFE treatment markedly ameliorated neuronal cell loss in the SCO group. Moreover, TAC also reversed the decreased neuronal cells (Fig. 2a). As shown in Fig. 2b and c, the number of Nissl-stained cells was markedly reversed by EEFE or TAC groups compared with the SCO group. To further confirm the neuroprotective effects of EEFE, we utilized the HT22 hippocampal cell line. The CCK assay was performed to establish whether EEFE was cytotoxic against HT22 cells. Cells were treated with 0, 12.5, 25, 50, or 100 μg/mL of EEFE for 24 h. EEFE treatment showed no significant effect on the viability of HT22 cells (Fig. 2d). Following these results, HT22 cells were treated with EEFE under non-toxic concentration and then exposed to H_2_O_2 _to induce the self-generation of free radicals as well as to produce oxidative stress in neuronal cells. Post H_2_O_2_ treatment, a significant reduction of cell viability was noticed. In contrast, EEFE or carvedilol (positive control) significantly inhibited H_2_O_2_-mediated cell death (Fig. 2e). We also determined the extent of cell death through an assay measuring the release of LDH into the media. Consistent with the results of the CCK assay, EEFE treatment was found to have a marked inhibitory effect on oxidative stress induced by H_2_O_2_ (Fig. 2f). Taken together, both in vivo and in vitro results indicated that EEFE had significant effects on neuronal cell protection.

### Antioxidant Activity of EEFE in SCO-Induced Memory Impairment Mice

**Fig. 3:**
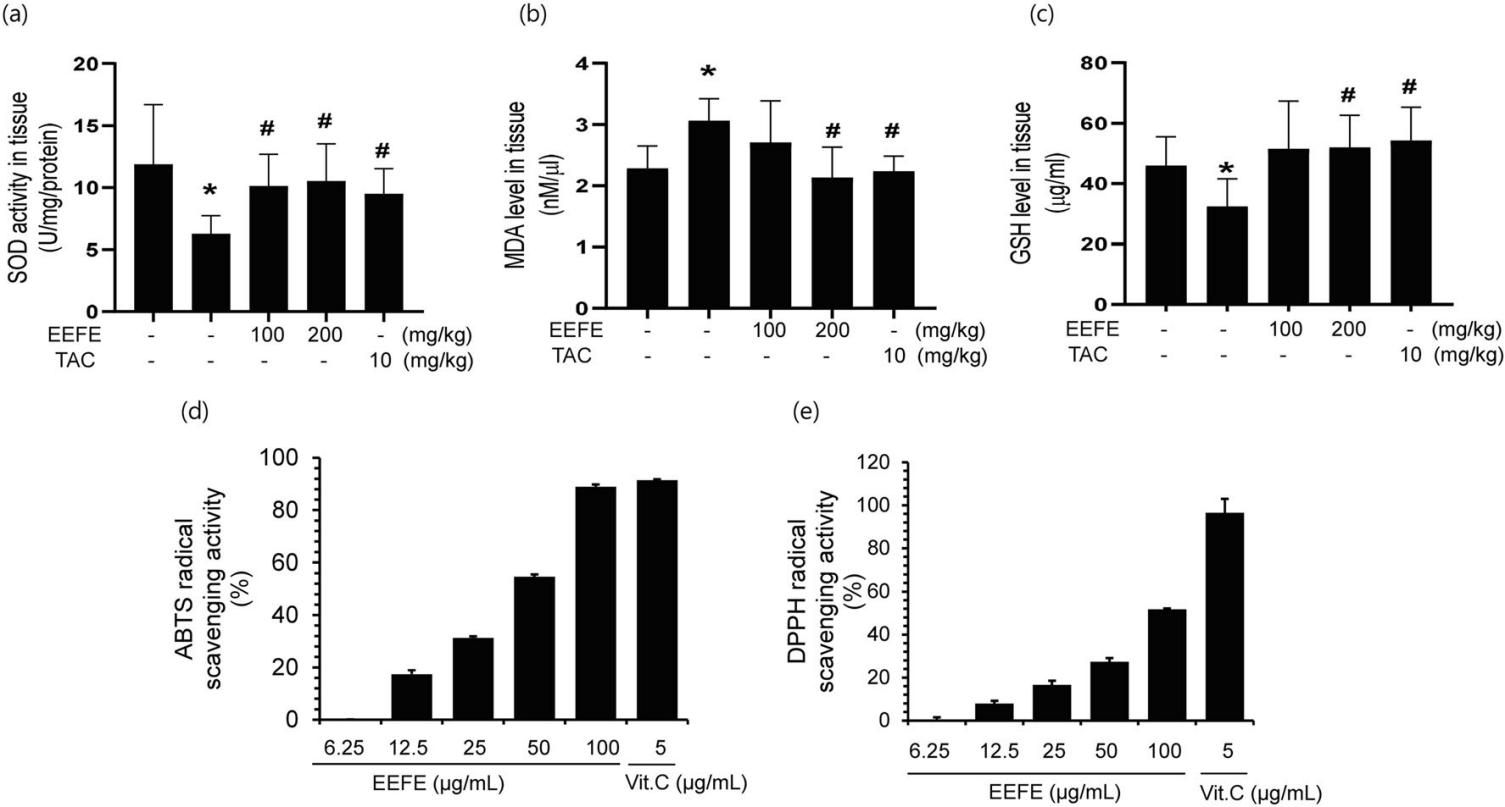
Antioxidant effects of EEFE in SCO-induced memory impairment mouse brains and hydrogen peroxide-treated neuronal cells. **a**–**c** Levels of intracellular antioxidant enzymes SOD (**a**), MDA (**b**), and GSH (**c**) were measured using supernatants from the brain lysates. Absorbance of the samples was read at 490, 540, and 405 nm, respectively. TAC was used as a positive control. Data are presented as the mean ± SEM (*n*=5). ^*^p<0.05 vs NOR group and ^#^*p*<0.05 vs SCO group. **d**, **e** Free radical scavenging activity assays were carried out for ABTS (**d**) and DPPH (**e**). Vit. C was used as a positive control. Data are presented as the mean ± SEM (*n*=3). EEFE, ethanol extracts of *Ficus erecta*; NOR, normal; SCO, scopolamine; TAC, tacrine; Vit. C, vitamin C; SOD: superoxide dismutase; MDA: malondialdehyde; GSH: glutathione

Furthermore, we assessed the effects of EEFE on antioxidant factors such as SOD, GSH levels, and contents of MDA in SCO-induced memory impairment mouse brain tissues. The EEFE group had significantly enhanced antioxidant SOD activity and GSH level and also showed decreased antioxidant MDA level compared to the SCO group (Fig. 3a–c). These results suggested that EEFE could have a protective effect against SCO-induced dysfunction related to the oxidative stress system in the brain tissue. ROS is associated with the cognitive dysfunction shown in the SCO-induced memory impairment animal model [32]. We further confirmed the antioxidant effects of EEFE by assessing radical scavenging activity using in vitro ABTS and DPPH assays. EEFE markedly increased the scavenging effects on the ABTS and DPPH radicals (Fig. 3d and e) in a concentration-dependent manner. We used vitamin C as a positive control of antioxidant [33].

### Effect of EEFE on the Nrf2/HO-1 Signaling in SCO-Treated Memory Impairment Mice

**Fig. 4:**
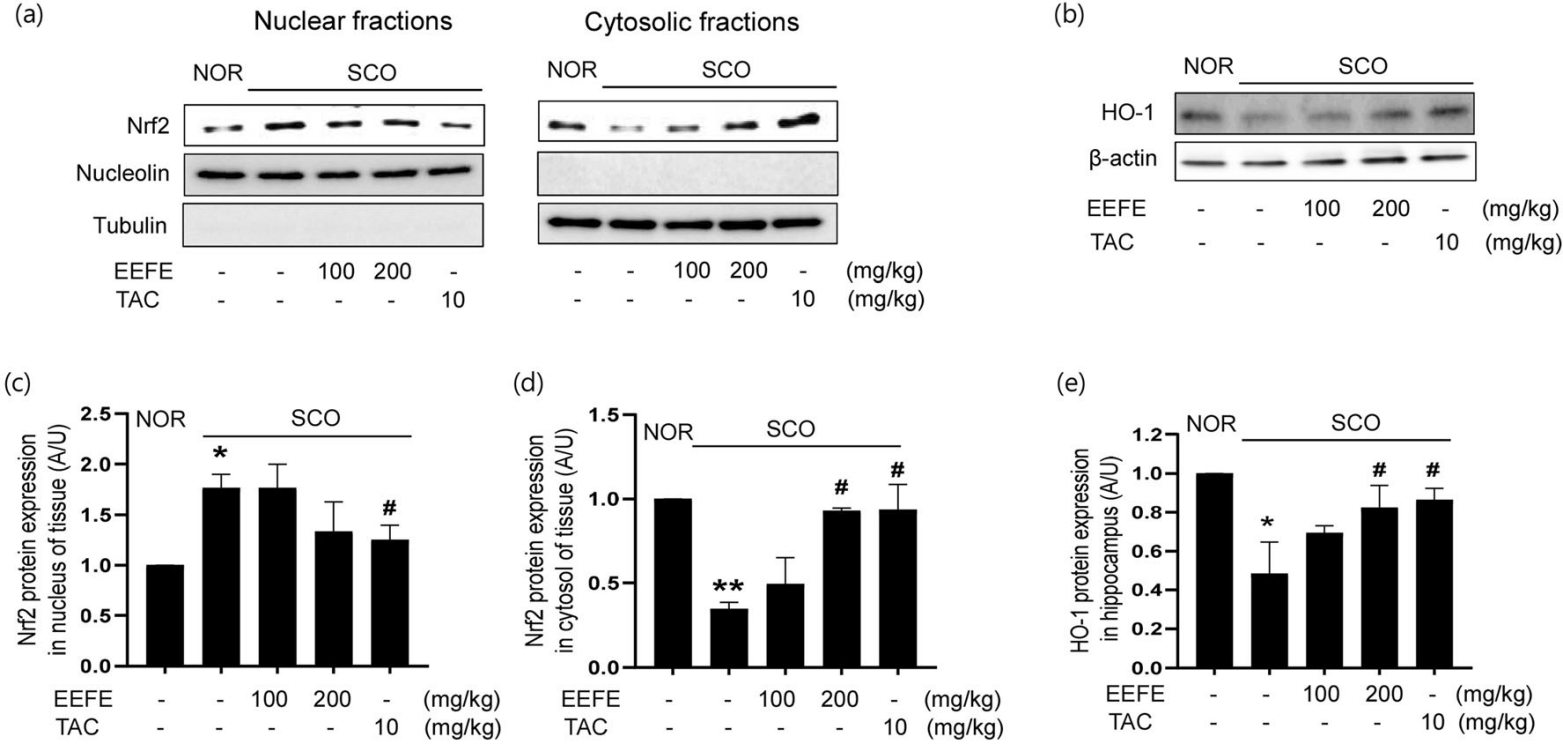
Effects of EEFE on the protein expressions of HO-1 and Nrf2 in SCO-induced memory impairment mouse brains. Western blotting was performed to determine the levels of Nrf2 and HO-1. **a**, **b** Representative photographs show the immunoblots for Nrf2 in nuclear (left) and cytosolic (right) fractions (**a**), and HO-1 in whole-brain tissues (**b**). **c**–**e** Bar graphs represent a quantitative analysis of relative band intensities of the Nrf2 in the nucleus (**c**) and cytosol (**d**), and HO-1 in whole-brain tissues (**e**) compared with NOR. Expression levels were normalized to β-actin for whole lysates, tubulin for cytosol, or nucleolin for the nucleus. TAC was used as a positive control. Data are presented as the mean ± SEM (*n=*5). ^**^p<0.01 vs NOR group, ^#^*p*<0.05 or ^##^*p*<0.01 vs SCO group. EEFE, ethanol extracts of *Ficus erecta*; NOR, normal; SCO, scopolamine; TAC, tacrine; Nrf2: nuclear factor erythroid-2-related factor 2; HO-1: heme oxygenase 1

Engaging Nrf2/HO-1 antioxidant responsive element signaling pathway is crucial in the defense mechanisms against nerve cells from oxidative stress [34]. Hence, we evaluated the effects of EEFE on the Nrf2/HO-1 pathway in SCO-induced memory impairment mice. As shown in Fig. 4a, Nrf2 expression was increased in nuclear fractions and decreased in cytosolic fraction in the SCO group, indicating nuclear translocation of Nrf by SCO injection. In contrast, cytosolic Nrf2 was increased in the EEFE group compared with the SCO group. Nuclear Nrf2 level was decreased in the EEFE group compared with the SCO group. We further measured the effect of EEFE on the expression of HO-1, an antioxidant enzyme. EEFE treatment markedly promoted the expression of HO-1 in brain tissue lysates compared to the SCO group (Fig. 4b). Quantitative analyses of band intensities of Nrf2 and HO-1 expression are shown in Fig. 4c–e.

### Effects of EEFE on Cholinergic System Dysfunction and Expression of Neuroprotective Factors in SCO-Treated Memory Impairment Mice

**Fig. 5:**
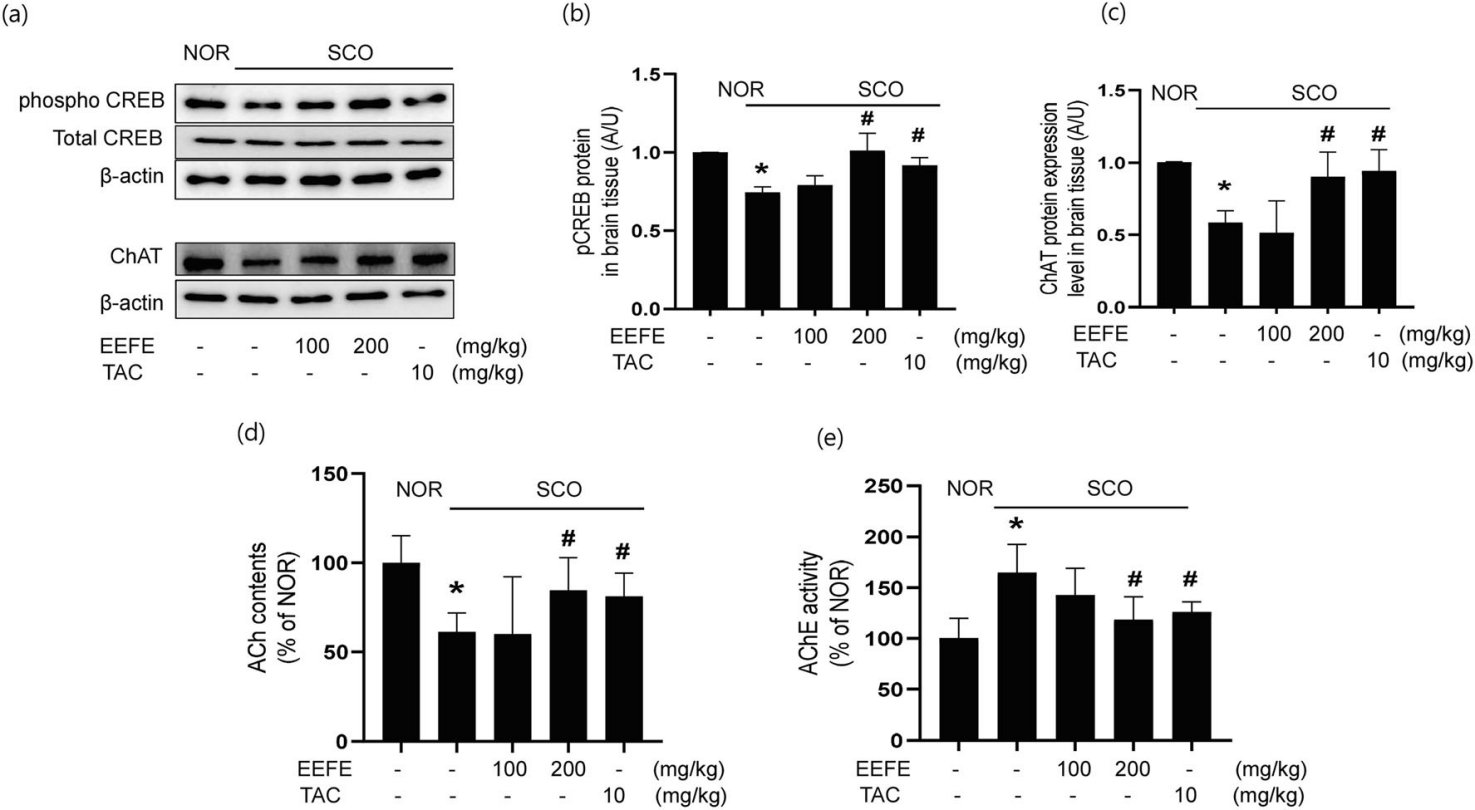
Effects of EEFE on the cholinergic system in SCO-induced memory impairment mouse brains. **a**–**c** Brain tissue lysates were applied to western blotting for detecting CREB phosphorylation (**b)** and ChAT expression (**c**). The protein levels were normalized to total CREB for pCREB and β-actin for ChAT. TAC was used as a positive control. Ach contents (**d**) and AChE activity (**e**) were measured in the brain tissue using an ACh and AChE activity assay kit (US Biomax Inc., CA, USA). Data are presented as the mean ± SEM (*n*=5). ^*^p<0.05 vs NOR group, ^#^*p*<0.05 vs SCO group. EEFE, ethanol extracts of *Ficus erecta*; NOR, normal; SCO, scopolamine; TAC, tacrine; CREB: cAMP response element-binding protein; ChAT: choline acetyltransferase; Ach: acetylcholine; AChE: acetylcholinesterase

Cholinergic system and neuroprotective factor pCREB are critical factors associated with formation of retention of existing memory [35]. To explore the possible mechanisms underlying the improving effects of EEFE on memory impairment induced by SCO, the levels of proteins associated with neuroprotection and cholinergic system were assessed. pCREB level was decreased by SCO-induced neurotoxicity (Fig. 5a and b). In contrast, EEFE or TAC treatment ameliorated SCO-mediated reduction of pCREB levels in the brain lysates. Furthermore, SCO treatment resulted in a considerable decrease in ChAT expression (Fig. 5c) and ACh content (Fig. 5d) and an increase in AChE activity (Fig. 5e) in the brain tissues. However, the EEFE or TAC treatment markedly inversed the effects on cholinergic system dysfunction by SCO injection.

### HPLC Determination of the Three Standard Compounds in EEFE

**Fig. 6:**
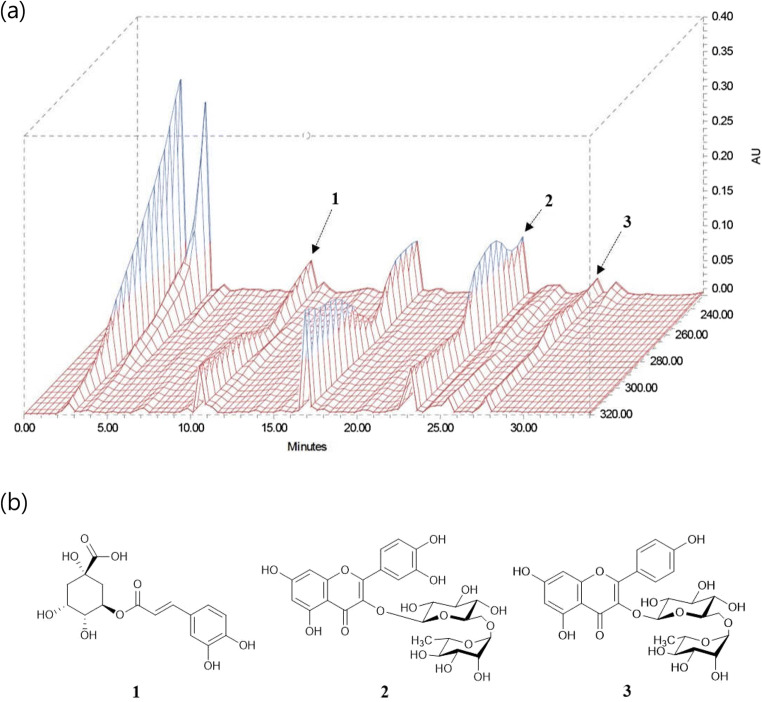
Three-dimensional HPLC chromatogram of EEFE (**a**) and the chemical structures of three standard compounds (**b**). 1: chlorogenic acid, 2: rutin, 3: kaempferol-3-O-rutinoside

Established HPLC method was used for the simultaneous determination of the three standard compounds in EEFE. The chromatograms indicated good separation using mobile phases consisting of 0.1% (v/v) aqueous acetonitrile and TFA. The UV wavelengths for the detection of the compounds were 320 nm for chlorogenic acid, and 260 nm for rutin and kaempferol-3-O-rutinoside. The three standard compounds were resolved within 28 min. The retention times of chlorogenic acid, rutin, and kaempferol-3-O-rutinoside were 10.54, 23.09, and 27.56 min, respectively. Three-dimensional HPLC chromatograms of the EEFE and chemical structures of three standard compounds are presented in Fig. 6a and b, respectively.

## Discussion

In this study, we evaluated that EEFE could protect against SCO-induced memory impairment in a mouse model. The results indicate that EEFE administration recovered memory impairment by inhibiting cholinergic system dysfunction and oxidative stress in SCO-induced memory impairment mice. In addition, EEFE had neuroprotective effects by promoting Nrf2/HO-1 pathway against SCO-induced oxidative stress and damage in the brain.

Memory and cognitive dysfunction are the salient hallmarks of AD [4, 5]. Many studies suggested that pathological processes of AD lead to regional neuron damage in the brain [36–38]; however, the precise mechanisms of AD pathogenesis have not been fully understood. In the cognitive function–related forebrain area, oxidative stress causes damage that manifests as neuronal death and neurodegeneration. Many preclinical animal studies and clinical trials have regarded oxidative stress and cholinergic system dysfunction as indicators of neuronal injury [4, 39–41]. Therefore, changes of cholinergic function in the forebrain are pursued as the goal for target for the treatment of AD syndromes [42].

SCO, typical acetylcholine receptor antagonist, disturbs memory and learning function [29]. SCO-induced memory impairment is a valid model for preclinical and clinical phases of drug trials in humans and animals that interferes with oxidative stress and the cholinergic system [10, 29]. Our data show the memory-enhancing effects of EEFE treatment in SCO-induced memory impairment mice using the Y-maze test and PAT, useful tools for the estimation of cognitive deficit [10, 28]. EEFE treatment prolonged the spontaneous alternation and step-through latency time, indicating the ameliorating effects of EEFE on the memory impairment induced by SCO injection.

Oxidative stress plays a critical role in the pathogenesis of AD. Increasing levels of oxidative stress in preclinical and clinical studies during the latent period of the disease lead to a sudden onset of symptoms of cognitive deficit [43, 44]. Levels of SOD and GSH in the antioxidant defense system are markedly reduced in SCO-induced memory impairment animals and human AD patients [42, 45]. The present study show the neuroprotective effects of EEFE against oxidative stress induced by SCO in mice and H_2_O_2_ in neuronal cells. Furthermore, EEFE administration enhanced antioxidant activities by increasing antioxidant enzyme SOD and levels of antioxidant GSH and reducing the contents of MDA in the brain tissues. Moreover, EEFE dramatically strengthened the scavenging activity against ABTS and DPPH radicals. The exhaustion of endogenous antioxidant enzymes such as SOD results in the overproduction of ROS that can directly cause cellular oxidative damage [46]. ROS leads to the process of lipid peroxidation in neuronal degeneration of the brain and reduces the capacity of the antioxidant defense system [43, 45, 47]. Thus, protective effects against neuronal death and oxidative damage in neurodegenerative diseases are important in the discovery of new therapeutic drugs or supplements.

Molecular mechanisms of oxidative stress–induced cell injury in neurodegeneration have been investigated. One of the molecular events is the increase of ROS production via stimulating oxidative stress-mediated Nrf2/HO-1 pathway [48, 49]. Nrf2 is a redox-sensitive transcription factor that is induced in the brain flowing toxic levels of stress and mediates the induction of detoxifying/antioxidant enzymes, such as HO-1. The Nrf2/HO-1 signaling pathway plays a pivotal role in neuroprotection in neurodegenerative disorders, including AD. Interestingly, numerous studies have indicated that natural products have potential to target oxidative stress related to the Nrf2/HO-1 pathway in a SCO-induced cognitive impairment model [49–51]. In the present study, EEFE exerted neuroprotective effects by upregulating HO-1 expression through activation of Nrf2. Therefore, we suggest that EEFE protects neuronal cells against SCO-induced neurotoxic oxidative stress via regulating the Nrf2/HO-1 pathway.

Prolonging the release of ACh into the synaptic clearance has been used as a means of enhancing cholinergic function in AD. SCO is an anticholinergic agent that provokes the muscarinic cholinergic receptors [52]. SCO injection markedly diminishes activation of the cholinergic system and cognitive function, as indicated by decreasing ACh and increasing AChE activity along with decreased ChAT activity, resulting in neuronal cell death under oxidative stress in the forebrain [29, 45]. Our results show that EEFE treatment reversed SCO-induced cholinergic dysfunction by decreasing Ach contents and ChAT expression levels, and increasing AChE activity in the forebrain. These results suggest that the memory-enhancing effects of EEFE on SCO-induced memory impairment could be explained by their modification of the cholinergic system. Besides, EEFE treatment ameliorated CREB phosphorylation in the forebrain tissue of SCO-induced mice. CREB, one of the major regulators of neurotrophin responses, is implicated inprotection, and development, acquisition, and consolidation of memory in the nerve system [53]. CREB also stimulates neuronal cell survival and activates neuroprotection by excessive production of ROS [54]. Taken together, our results indicate that EEFE exerts memory-enhancing effects through the regulation of the cholinergic system and phosphorylation of CREB.

Interest in the potential of phytochemicals to enhance learning, cognitive ability and memory as modulators of brain function has been on the rise, recently. A possible relationship between the intake of polyphenols, one of the classes of phytochemicals, and prevention of AD has been reported [55–57]. Previous studies also indicated that dietary ingestion of rich polyphenolic compounds from plants suspended the onset of dementia involved with AD [12, 58]. In our study, we confirmed that EEFE has three major compounds— rutin, chlorogenic acid, and kaempferol-3-rutinoside—by HPLC analysis. These flavonoids are abundantly presented in various natural plants for a long time and are linked to the ability of flavonoids to involve in memory function, including synaptic potentiation and plasticity [55, 59]. In previous reports, chlorogenic acid and rutin were revealed to have strong antioxidant activities associated with free radical scavenging and positive effects in ameliorating cognitive dysfunction through different behavioral tests in rodent models [60, 61]. Kaempferol-3-rutinoside, a glucosidic derivative of kaempferol, has beneficial effects on cytotoxic and anti-inflammatory activity in numerous carcinoma cells in vitro [56]. Several studies showed that kaempferol-3-rutinoside has protective effects against multi-infarct dementia and cerebral ischemic damage [62, 63]. These reports may closely associate with the favorable effects of EEFE composing the three major phytochemicals on anti-neurodegeneration.

## Conclusions

There are several AD medications, such as cholinesterase inhibitors and NMDA receptor antagonists. However, they do not have curing effects, and their unexpected side effects can lead to limitations in their use in patients. The use of natural product supplements with non-toxic, multi-targeting, and high efficacies may delay the progression of AD. Our current study is providing evidence that *F. erecta* Thunb leaves have a potent neuroprotective effect. EEFE can improve the cognitive deficits in SCO-injected mice. The effects of EEFE are mainly related to its excellent antioxidant activity via regulating the Nrf2/HO-1 pathway and associated with modulating neuronal cell damage via activation of the cholinergic system and CREB signaling. We conclude that EEFE may be a promising candidate for the prevention or treatment against AD or AD-involved neurodegenerative diseases.

## Data Availability

The data supporting the findings in this research are provided within the manuscript.

## Declarations

All animal experimental procedures in this study were performed in accordance with the ethical standards of the institution or approved by the institutional animal care and use committee.
